# Automated quality assurance of rigid brain CT/MR image registration using a 3D convolutional neural network

**DOI:** 10.1002/acm2.70828

**Published:** 2026-09-28

**Authors:** Zeinab A. Chahrour, Nour F. Obeid, Ali El‐Zaart, Wassila EL Kanawati, Abbas Mkanna, Rida Assaf, Bilal H. Shahine

**Affiliations:** ^1^ Department of Physics Beirut Arab University Beirut Lebanon; ^2^ Department of Computer Science American University of Beirut Beirut Lebanon; ^3^ Department of Mathematics and Computer Science Faculty of Sciences Beirut Arab University Beirut Lebanon; ^4^ Department of Radiation Oncology American University of Beirut Medical Center Beirut Lebanon

**Keywords:** 3D convolutional neural network, CT/MR rigid image registration, Registration quality assurance

## Abstract

**Background:**

Accurate CT/MR registration is important in stereotactic brain radiotherapy, where small spatial errors may affect target localization and treatment planning. In many clinical settings, registration quality is still assessed primarily through manual visual inspection. However, time and workflow constraints may limit the consistency and depth of manual evaluation.

**Purpose:**

This study aimed to develop a 3D convolutional neural network (3D CNN)‐based quality assurance (QA) method for rigid brain CT/MR registration using paired sub‐volumes and a registration‐level mean probability score (*μ*). The proposed method classifies local CT/MR registration quality, while the final registration‐level assessment is based on *μ* across the sampled sub‐volumes.

**Methods:**

The study included 209 patients. Of these, 202 comprised the primary cohort and were divided patient‐wise into 128 training, 33 validation, and 41 test patients. For each patient, 25 skull‐based CT/MR sub‐volumes of 42 ×  42 ×  42 voxels were used as a two‐channel CNN input. High‐quality examples were obtained from clinically approved registrations, while low‐quality examples were generated using rigid perturbations of the complete MR volume. The mean predicted probability across the 25 sub‐volumes was used as the registration‐level score *μ*, and the validation‐derived threshold of 0.5871 was fixed for testing. Seven additional patients, separate from the 202‐patient cohort, were evaluated after model development and threshold selection were completed using registrations before and after physician correction.

**Results:**

On the held‐out test set, patch‐level accuracy was 85.90%, with an ROC AUC of 0.929 and average precision of 0.920. At the registration level, accuracy was 97.56% and ROC AUC was 0.999. All 41 low‐quality registrations were correctly flagged, while 39 of 41 high‐quality registrations were correctly accepted. In the additional clinical evaluation, all seven initial registrations requiring correction were classified as low quality, while six of seven corrected registrations were classified as high quality, resulting in an overall accuracy of 92.86%.

**Conclusions:**

The proposed method combined local CNN predictions into a registration‐level *μ* score and showed strong performance on held‐out and clinical cases. It may provide a quantitative screening measure for rigid brain CT/MR registration QA while maintaining clinical review as the final decision step.

## 1 INTRODUCTION

Image registration plays an important role in treatment planning and image‐guided treatment delivery. It is used to determine a geometric transformation that aligns a moving image with a fixed reference image by matching corresponding anatomical structures. In radiotherapy, image registration is used for image segmentation, adaptive treatment planning, and image‐guided radiotherapy. Rigid image registration (RIR) preserves the distance between all image points and consists of translations and rotations along the three spatial axes.^1^

In clinical radiotherapy workflows, computed tomography (CT) is routinely used for treatment planning and provides a three‐dimensional representation of patient anatomy. Hounsfield unit (HU) values from CT scans can be calibrated to generate electron density maps for dose calculation. Magnetic resonance imaging (MR) is frequently used alongside CT to improve soft‐tissue visualization^2^ and allows more accurate target and structure delineation because of its superior soft‐tissue contrast.^3^ Consequently, CT and MR images are commonly combined for diagnosis and treatment planning.

Accurate CT/MR registration remains clinically challenging and has increased interest in methods for evaluating registration quality.^4^ In many clinical settings, registration quality is still assessed primarily through manual visual inspection. However, time and workflow constraints may limit the consistency and depth of manual evaluation. Automated quality assurance (QA) methods may therefore help support the clinical review process by identifying registrations that require closer inspection.^5^

High registration accuracy is particularly important in stereotactic treatments such as stereotactic radiosurgery (SRS) and stereotactic radiation therapy (SRT), where even small spatial errors may affect target localization and treatment delivery. The AAPM TG‐101 report emphasizes the importance of precise image‐guided localization and spatial accuracy in stereotactic radiotherapy workflows.^6^

Convolutional neural networks (CNNs) are deep learning models that learn hierarchical image features through convolution operations.^7^ CNNs have been widely applied in radiology and medical image analysis for tasks including classification,^8^, ^9^ segmentation,^10^ and detection.^11^

Although several studies have investigated image registration and registration error estimation, many existing approaches focus on deformable registration, single‐modality CT images, or improving the registration process itself. In contrast, fewer studies have investigated automated quality assessment of clinically performed rigid CT/MR registrations in brain radiotherapy planning, where the final decision is typically made after visual review. There remains a need for a QA screening method that can support the evaluation of existing CT/MR registrations and help identify cases that may require additional manual assessment.

The aim of this study was to develop and evaluate a supervised 3D CNN‐based QA method for rigid brain CT/MR registration using paired skull‐based CT/MR sub‐volumes. The proposed method classifies local CT/MR registration quality, while the final registration‐level assessment is based on the mean predicted probability (*μ*) across the sampled sub‐volumes. An additional evaluation was performed using clinical registrations that required physician correction during routine review. We hypothesized that local CNN‐based predictions, when summarized using *μ* at the registration level, can provide a useful screening score for identifying rigid CT/MR registrations that may require closer manual review.

Several methods have been proposed to quantify image registration errors. These methods are generally divided into unsupervised and supervised approaches. In unsupervised approaches, quality assessment is based on data analysis without using a learning model. Crum et al.^12^ introduced a method to estimate errors in voxel‐based registration using the Gaussian scale space of the post‐registration residual image. This method cannot localize errors spatially and is limited to rigid transformations. Datteri et al.^13^ proposed a registration quality assessment method based on a registration circuit and reported a correlation with target registration error (TRE). Saygili et al.^14^ introduced a method that did not use ground truth and was independent of the registration algorithm. Their approach extracted voxel‐level image features to estimate local registration errors.

In supervised approaches, models are trained using datasets with reference quality labels. Neylon et al.^15^ developed a three‐layer neural network to estimate the accuracy of deformable image registration (DIR) using a simulated head‐and‐neck dataset. Their method achieved approximately 95% accuracy within a single voxel. Galib et al.^5^ presented a 3D CNN framework for estimating DIR errors in lung CT scans using a Registration Error Index (REI). They reported an AUC‐ROC of 0.882, with errors close to 11% of the true value.

Walluscheck et al.^16^ used a multi‐atlas registration approach to align MR atlases with CT brain scans. They reported high Dice values for manual segmentation, while CNN‐based segmentation gave slightly lower values.

Recent studies have also used deep learning for medical image registration. Fu et al.^17^ reviewed deep learning‐based registration methods and described several convolutional and generative approaches. Lei et al.^18^ proposed a dual‐feasible neural network for forward and backward registration, and Ma et al.^19^ introduced a global‐local transformation model to improve deformable registration. These studies mainly focused on improving the registration process itself. In contrast, the present study focuses on assessing the quality of an existing rigid CT/MR registration after clinical registration has already been performed, which represents the main gap addressed in this work.

## 2 MATERIALS AND METHODS

### 2.1 Dataset

This study included 209 patients in total. The primary cohort consisted of paired brain CT and MR scans from 202 patients who underwent stereotactic radiosurgery (SRS) or stereotactic radiation therapy (SRT). Seven additional patients, separate from this primary cohort, were used for the independent clinical evaluation described below. CT images were acquired at the American University of Beirut Medical Center (AUBMC) using a multi‐slice helical CT scanner (SOMATOM Sensation, Siemens Healthineers, Erlangen, Germany) with a standard radiotherapy planning protocol of 120 kV and 400 mAs. Images were reconstructed with a slice thickness of 1.5 mm, an H10s reconstruction kernel, an in‐plane pixel spacing of 0.927 ×  0.927 mm, and a reconstructed field of view of 475 mm.

Of the 202 MR datasets, 153 were acquired at AUBMC and 49 were obtained from external institutions and accepted for stereotactic treatment planning at AUBMC. MRI scans acquired at AUBMC were obtained using a Philips BlueSeal XE 1.5 T scanner with a 3D T1‐weighted gadolinium‐enhanced sequence (TR = 7.1 ms, TE = 3.23 ms). All external MRI datasets also consisted of 3D T1‐weighted gadolinium‐enhanced images. Although the external MRI scans were acquired using different scanners, the same type of stereotactic post‐contrast sequence was used. Detailed scanner‐specific acquisition parameters were not uniformly available for the external datasets.

Data were partitioned on a patient‐wise basis to prevent data leakage. Of the 202 patients, 128 were assigned to the training set, 33 to the validation set, and 41 to the held‐out test set. The validation set was used for model selection and derivation of the registration‐level mean probability (*μ*) threshold, whereas the test set was kept separate and used only for final evaluation.

To minimize anatomical changes caused by disease progression or treatment effects, the interval between CT and MR acquisitions did not exceed one week according to the stereotactic treatment protocol. Cases with major post‐surgical anatomical changes, severe edema‐related deformation, severe metallic artifacts, or other image changes that prevented clinical use for stereotactic planning were not included.

This retrospective study was conducted in accordance with institutional ethical standards. All patient data were anonymized before analysis in accordance with patient privacy requirements.

#### 2.1.1 Rigid registration framework

Image registration was performed using Velocity 3.2.0 (Varian Medical Systems, Palo Alto, CA, USA). Initial registrations were performed automatically by selecting the primary and secondary images, setting the appropriate image contrast, and defining the region of interest for registration. Manual adjustments were applied when required to improve anatomical correspondence, and each registration result was reviewed and approved by a radiation oncologist. These clinically approved rigid CT/MR registrations were used as the high‐quality registration examples in this study.

For an additional independent clinical evaluation, seven patients who were separate from the 202‐patient primary cohort were identified during routine clinical review after model development and threshold selection had been completed. Their initial CT/MR registrations were not accepted by the reviewing physician and required manual adjustment. These pre‐correction registrations were retained as clinical low‐quality cases. After manual adjustment by the physician, the corrected and clinically approved registrations were retained as the corresponding high‐quality cases. The finalized model and the fixed validation‐derived *μ* threshold of 0.5871 were then applied to both registration conditions without further adjustment.

#### 2.1.2 Image preprocessing

All CT and MR DICOM series were converted to compressed NIfTI format (.nii.gz) using DicomRTTool, with SimpleITK used as a fallback when required. Before intensity preprocessing and sub‐volume extraction, CT and MR images were mapped onto a common physical reference grid derived from the CT geometry. The common grid used a voxel spacing of 0.8 ×  0.8 ×  1.0 mm in the x, y, and z directions, respectively, while preserving the CT physical extent, origin, and image orientation. Linear interpolation was used for resampling.

The CT image and the clinically registered MR image were each resampled once onto the same common reference grid. This ensured that corresponding CT and MR locations were defined in the same physical coordinate system before landmark selection and patch extraction.

For CT preprocessing, the resampled CT volume was center‐cropped in the axial plane to 212 ×  212 voxels while preserving the full z‐extent. No additional axial resizing was performed. Mild Gaussian smoothing with *σ* = 1.0 was then applied. CT intensities were clipped to the range −1000 to 3000 HU and linearly scaled to [0,1] using the fixed transformation ((HU+1000)/4000).

For MR preprocessing, the resampled MR volume was center‐cropped to the same 212 ×  212 axial dimensions. Voxels with intensities between 0 and 10 were set to zero. The remaining positive MR intensities were normalized using the 1st and 99th percentiles calculated for each volume and were clipped to [0,1].

A cranial skull mask was generated from the cropped CT image before Gaussian smoothing and intensity normalization. Bone voxels were identified using a threshold of 700–3000 HU. Three‐dimensional connected components smaller than 5000 voxels were removed, followed by binary morphological closing with a radius of 2 voxels. The inferior 15% of the image volume was excluded from landmark sampling to reduce inclusion of the mandible and cervical vertebrae and to focus sampling on the skull regions relevant to rigid brain CT/MR registration.

#### 2.1.3 Sub‐volume extraction

A landmark‐based sub‐volume extraction strategy was used instead of full‐volume training to reduce memory requirements while providing spatially distributed samples across the cranial anatomy.

For each patient, exactly 25 landmark centers were sampled from the CT‐derived cranial skull mask. Landmark locations were distributed across the superior‐inferior extent of the skull and were required to be separated by a minimum Euclidean distance of 10 voxels. Landmark locations were also restricted to ensure that the complete sub‐volume remained within the image boundaries. Because the minimum distance between landmark centers was smaller than the sub‐volume dimension, partial overlap between neighboring sub‐volumes was allowed. A maximum of 100,000 sampling attempts was allowed when additional valid landmarks were required.

Each CT landmark was converted to its physical coordinate and mapped to the corresponding MR voxel location. At each landmark, paired CT and MR sub‐volumes of size 42 ×  42 ×  42 voxels were extracted. The MR and CT sub‐volumes were stacked as two separate input channels for the CNN.

The paired sub‐volume preparation and two‐channel input configuration are illustrated in Figure 1.

**FIGURE 1:**
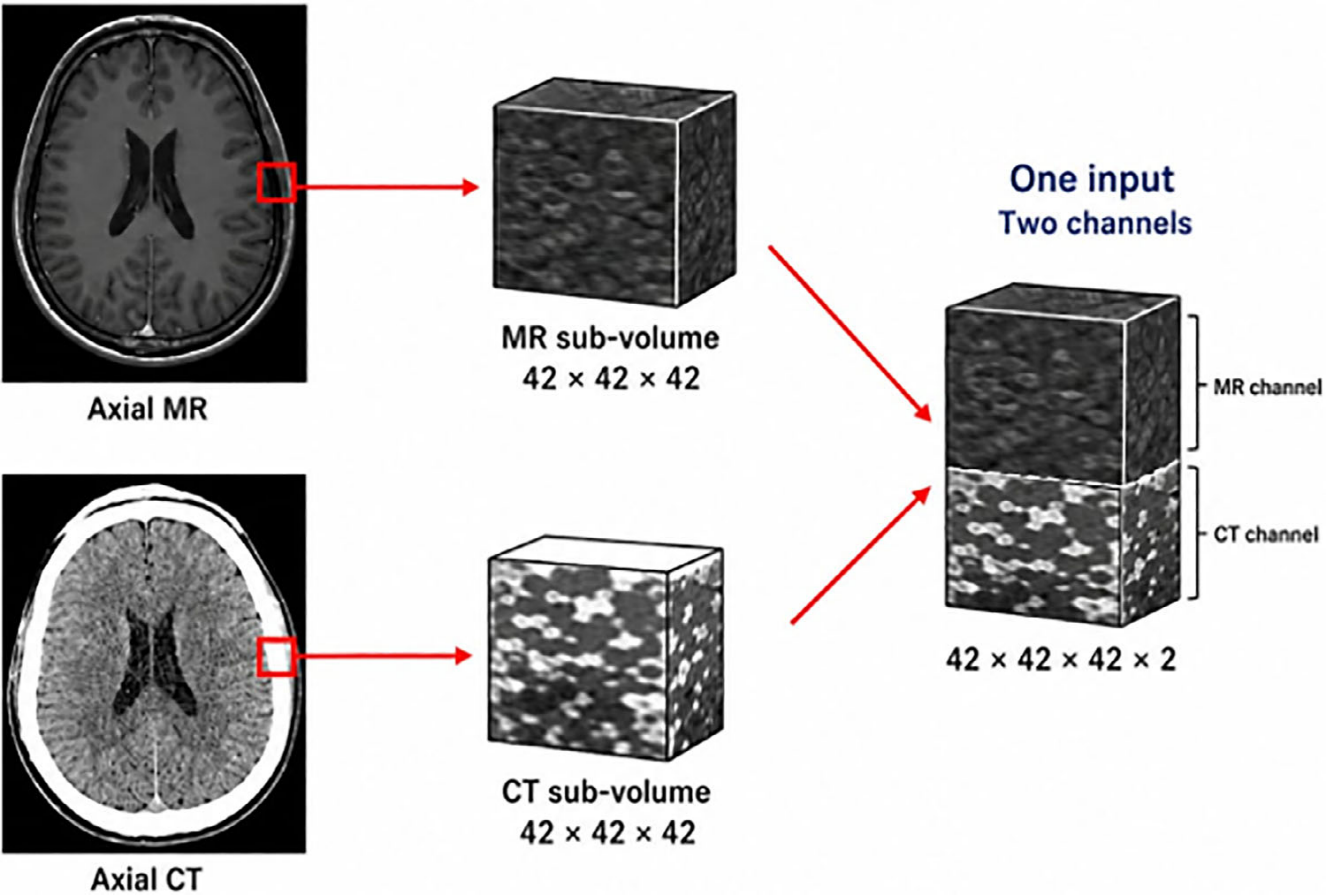
Input sample preparation. 3D MR and CT images were used to extract paired sub‐volumes from the same spatial location. The paired MR and CT sub‐volumes were then arranged as one two‐channel input to the neural network, with the MR sub‐volume as the first channel and the CT sub‐volume as the second channel.

#### 2.1.4 Negative example generation

Low‐quality registration examples were generated by applying one six‐degree‐of‐freedom rigid perturbation to the MR image for each patient, with rotations and translations independently sampled within ± 5° and ± 5 mm, respectively.

The perturbation was applied around the physical center of the MR image using the SimpleITK Euler3DTransform.^20^ Importantly, the rigid perturbation was incorporated directly into the resampling of the original MR image onto the same CT‐derived common reference grid. Therefore, both the high‐quality and low‐quality MR images underwent a single linear resampling operation before intensity preprocessing and patch extraction.

The same patient‐specific perturbation was applied to the complete MR volume, and all 25 low‐quality patches from that patient were extracted from this perturbed volume. At every landmark, the clinically registered MR patch paired with the corresponding CT patch was labeled as high quality (1), whereas the perturbed MR patch paired with the same CT patch was labeled as low quality (0). Thus, each patient contributed 25 high‐quality and 25 low‐quality CT/MR pairs.

#### 2.1.5 Final dataset composition

All 202 patients produced the required 25 landmark locations. Each patient therefore contributed 50 CT/MR sub‐volume pairs: 25 high‐quality pairs and 25 corresponding low‐quality pairs. The final dataset contained 10,100 two‐channel CT/MR samples, consisting of 5,050 high‐quality and 5,050 low‐quality samples.

Patient‐wise splitting resulted in 128 patients and 6,400 samples for training, 33 patients and 1,650 samples for validation, and 41 patients and 2,050 samples for testing. Each split contained equal numbers of high‐ and low‐quality samples, and no patient appeared in more than one subset. The validation set was used for model selection and derivation of the registration‐level *μ* threshold, while the test set remained separate for final model evaluation.

### 2.2 Model architecture

In this work, we kept the model simple and efficient to fit the available GPU resources while still capturing the needed 3D features from the data.

The model had three Conv3D layers with 32, 64, and 128 filters. Each layer employed a 3 × 3 × 3 kernel, followed by a rectified linear unit (ReLU) activation function and a MaxPooling3D layer with a pool size of 2 × 2 × 2. The input blocks corresponded to the preprocessed paired CT/MR sub‐volumes. Each input had a size of 42 ×  42 ×  42 voxels with two channels, with the MR sub‐volume in the first channel and the CT sub‐volume in the second channel. For model training, the input was arranged in channels‐last format as 42 ×  42 ×  42 ×  2. The extracted feature maps were then flattened and passed through four dense layers of 64 neurons each, using ReLU activation. A dropout of 10% was added after every dense layer. The model was trained to classify CT/MR registration pairs as high quality or low quality. Figure 2 shows the model architecture. Table 1 lists the main parameters, optimizer setup, and training details.

**FIGURE 2:**
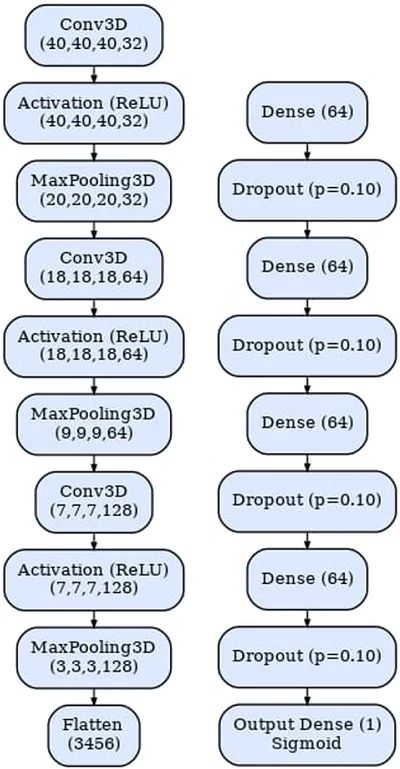
Architecture of the 3D CNN model. The input preparation is shown in Figure 1. The model includes three Conv3D blocks followed by dense layers and a sigmoid output.

**TABLE 1:** Summary of the proposed 3D CNN model architecture and training settings.

Item	Value
Input size	42 × 42 × 42 × 2
Conv3D filters	32, 64, 128
Kernel size	3 × 3 × 3
Max pooling	2 × 2 × 2
Dense layers	4 layers, 64 neurons each
Dropout	10% after each dense layer
Output layer	1 sigmoid neuron
Total parameters	512,225
Trainable parameters	512,225
Non‐trainable parameters	0
Optimizer	Adam
Learning rate	5 × 10^−^^4^
Loss function	Binary cross‐entropy
Training setup	Up to 20 epochs, batch size = 32, early stopping (patience = 5)
Hardware	NVIDIA A100 GPU, Google Colab

### 2.3 Model training and validation

The 3D CNN model was trained using the Adam optimizer with a learning rate of 5 ×  10^−^^4^ and binary cross‐entropy as the loss function for the binary classification problem. Training was performed for a maximum of 20 epochs with a batch size of 32 on an NVIDIA A100 GPU using Google Colab.

The patient‐wise data split was performed using GroupShuffleSplit with random_state = 42. First, 20% of the patients were assigned to the held‐out test set. The remaining patients were then split again, with 20% assigned to the validation set. This resulted in 128 patients for training, 33 patients for validation, and 41 patients for testing, corresponding to 6,400, 1,650, and 2,050 sub‐volume pairs, respectively. Each subset contained equal numbers of high‐ and low‐quality examples. Patient IDs were used as the grouping variable, and set‐based checks were applied to confirm that no patient appeared in more than one subset.

Model checkpointing was used to save the model with the highest validation accuracy. Early stopping with a patience of 5 epochs was also used, with restoration of the best‐performing weights. TensorBoard and CSV logging were used to follow the training process. Fixed random seeds were set for Python, NumPy, and TensorFlow.

During training, model performance was monitored using accuracy and loss. After training, classification performance was evaluated using accuracy, precision, recall, and F1‐score. These metrics were computed as:

(1) $$\mathrm{Classification}\;\mathrm{accuracy}\;=\frac{TP+TN\;}{TP+TN+FP+FN}\;$$

(2) $$F1-\mathrm{score}\;=2\;\times \frac{\mathrm{Precision}\;\times \mathrm{Recall}}{\mathrm{Precision}+\mathrm{Recall}}$$

(3) $$\mathrm{Precision}\;=\frac{\mathrm{TP}}{\mathrm{TP}+\mathrm{FP}}$$

(4) $$\mathrm{Recall}\;=\frac{\mathrm{TP}}{\mathrm{TP}+\mathrm{FN}\;}$$

where *TP*, *TN*, *FP*, and *FN* denote True/False positive and negative counts, respectively. In this binary classification setting, the positive class represents high‐quality registrations, and the negative class represents low‐quality registrations. Therefore, *TP* and *FN* quantify correctly and incorrectly identified high‐quality registrations, whereas *TN* and *FP* quantify correctly and incorrectly identified low‐quality registrations.

Accuracy, precision, recall, and F1‐score are commonly used supervised learning metrics in medical image‐analysis and machine‐learning studies.^21^, ^22^ Precision reflects the proportion of predicted high‐quality registrations that were truly high quality, while recall reflects the proportion of true high‐quality registrations correctly identified by the model. The F1‐score combines precision and recall into one value. These metrics were used to assess how well the model distinguished between high‐ and low‐quality CT/MR registration sub‐volumes.

### 2.4 Registration‐level mean probability (*μ*) and threshold selection

For registration‐level evaluation, the predicted probabilities from the sub‐volumes belonging to the same registration condition were combined into a single mean probability score, denoted as *μ*. For each high‐quality or low‐quality registration condition, *μ* was calculated as:

(5) $$\pi =\frac{1}{N}\sum_{i\;=1}^{N}\left(\mathrm{pi}\right)\;$$

where *N* is the number of extrated sub‐volumes for a given registration condition and *pi* is the predicted probability of the *i^th^* sub‐volume being high quality. Thus, higher *μ* values indicate greater model confidence that the CT/MR registration is high quality.

The decision threshold for *μ* was determined using only the validation set. For each validation patient, the clinically approved high‐quality registration and the corresponding synthetically perturbed low‐quality registration were treated as separate registration‐level cases. Receiver operating characteristic (ROC) analysis was performed using the resulting *μ* values, and the optimal threshold was selected using the Youden index. This analysis yielded a *μ* threshold of 0.5871.

The threshold was fixed and applied to the held‐out test set without further adjustment. Registration conditions with *μ* ≥ 0.5871 were classified as high quality, whereas those with *μ *< 0.5871 were classified as low quality and flagged for review.

## 3 RESULTS

The results focus on patch‐level classification performance and registration‐level evaluation using the mean predicted probability (*μ*). The validation set was used to select the *μ* threshold, and the fixed threshold was applied to the held‐out test set to evaluate registration quality. After restoration of the best‐performing model weights, the model achieved a training accuracy of 0.892 and a training F1‐score of 0.890. On the validation set, the model achieved an accuracy of 0.858 and an F1‐score of 0.861. The training and validation curves are shown in Figure 3.

**FIGURE 3:**
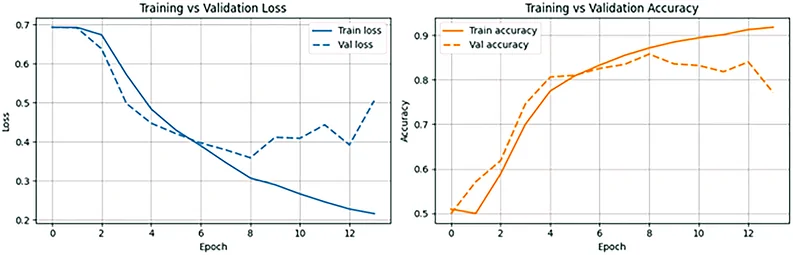
Training and validation curves of the 3D CNN model, showing loss on the left and accuracy on the right. Early Stopping was applied based on validation performance.

### 3.1 Validation‐based *μ* threshold selection

Registration‐level *μ* values were first calculated on the validation set to select the classification threshold. The validation set included 33 patients, with one high‐quality and one low‐quality registration condition for each patient, resulting in 66 registration‐level validation cases.

For the high‐quality validation cases, *μ* values ranged from 0.367 to 0.950, with a mean of 0.804. For the low‐quality validation cases, *μ* values ranged from 0.025 to 0.493, with a mean of 0.191. The individual registration‐level *μ* values for the validation set are reported in Table 2.

**TABLE 2:** Registration‐level mean probability (*μ*) values for the validation set.

Case No.	High *μ*	Low *μ*
**1**	0.95	0.32
**2**	0.84	0.15
**3**	0.71	0.33
**4**	0.82	0.31
**5**	0.82	0.13
**6**	0.78	0.05
**7**	0.92	0.24
**8**	0.89	0.20
**9**	0.89	0.08
**10**	0.84	0.17
**11**	0.87	0.02
**12**	0.81	0.44
**13**	0.86	0.03
**14**	0.69	0.20
**15**	0.59	0.11
**16**	0.87	0.18
**17**	0.77	0.11
**18**	0.94	0.25
**19**	0.87	0.18
**20**	0.77	0.15
**21**	0.89	0.14
**22**	0.84	0.14
**23**	0.63	0.22
**24**	0.76	0.18
**25**	0.83	0.08
**26**	0.78	0.49
**27**	0.89	0.30
**28**	0.83	0.16
**29**	0.93	0.11
**30**	0.37	0.15
**31**	0.66	0.30
**32**	0.83	0.12
**33**	0.79	0.26

*Note*: Values are rounded to two decimal places for presentation. Threshold selection and classification decisions were performed using the exact *μ* values. The validation‐derived *μ* threshold was 0.5871.

ROC analysis was performed using the registration‐level *μ* values. The validation AUC was 0.998. The Youden index selected a *μ* threshold of 0.5871. At this threshold, the validation accuracy was 98.48%, with a sensitivity of 96.97% for high‐quality registrations and a specificity of 100.00% for low‐quality registrations. The precision and F1‐score for the high‐quality class were 100.00% and 98.46%, respectively.

At the validation threshold, 32 of the 33 high‐quality cases were correctly accepted, while one high‐quality case was flagged for review. All 33 low‐quality cases were correctly flagged. The validation *μ* distribution and selected threshold are shown in Figure 4, and the corresponding threshold performance is summarized in Table 3. The threshold was then fixed and applied to the held‐out test set without further adjustment.

**FIGURE 4:**
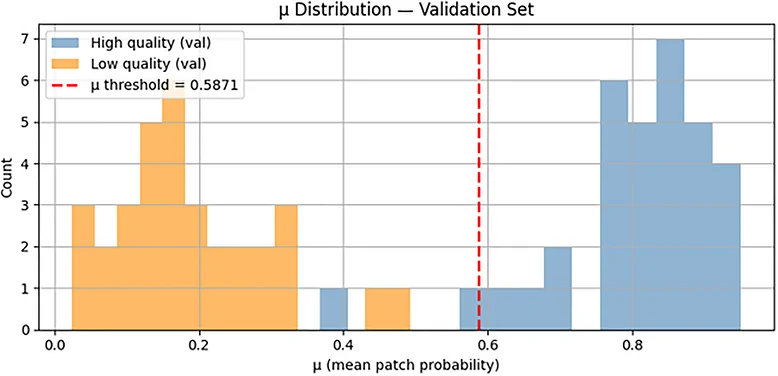
Distribution of registration‐level mean probability (*μ*) values in the validation set for high‐quality and low‐quality cases. The dashed vertical line indicates the selected *μ* threshold (0.5871).

**TABLE 3:** Validation‐based *μ* threshold performance summary.

Metric	Value
**Number of validation patients**	33
**Registration‐level cases**	66
**ROC AUC**	0.998
**Selected *μ* threshold**	0.5871
**Accuracy**	98.48%
**Precision for high‐quality class**	100%
**F1‐score for high‐quality class**	98.46%
**Sensitivity for high‐quality**	96.97%
**Specificity for low‐quality**	100%
**High‐quality correctly accepted**	32/33
**High‐quality flagged for review**	1/33
**Low‐quality correctly flagged**	33/33
**Low‐quality incorrectly accepted**	0/33

### 3.2 Held‐out test results

The held‐out test set included 41 patients and 2,050 CT/MR sub‐volume pairs, consisting of 1,025 high‐quality and 1,025 low‐quality samples. At the patch level, the model achieved an accuracy of 85.90%, with an ROC AUC of 0.929 and an average precision of 0.920. The F1‐scores were 0.860 for the high‐quality class and 0.858 for the low‐quality class. The corresponding patch‐level ROC and precision–recall curves are shown in Figure 5.

**FIGURE 5:**
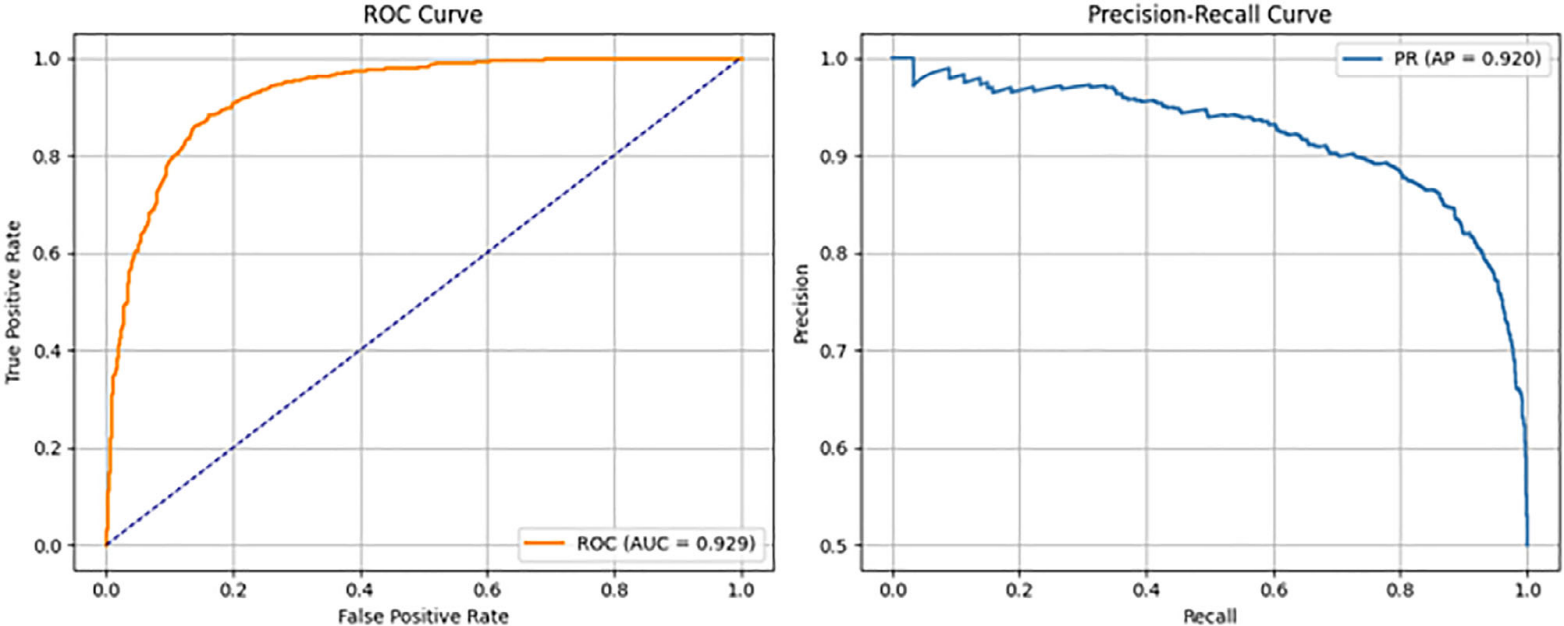
Patch‐level performance of the 3D CNN on the held‐out test set. Left: receiver operating characteristic (ROC) curve with an AUC of 0.929. Right: precision‐recall curve with an average precision (AP) of 0.920.

Among the 1,025 high‐quality sub‐volumes, 886 were correctly classified as high quality, and 139 were classified as low quality. Among the 1,025 low‐quality sub‐volumes, 875 were correctly classified as low quality, and 150 were classified as high quality. The patch‐level confusion matrices are shown in Figure 6.

**FIGURE 6:**
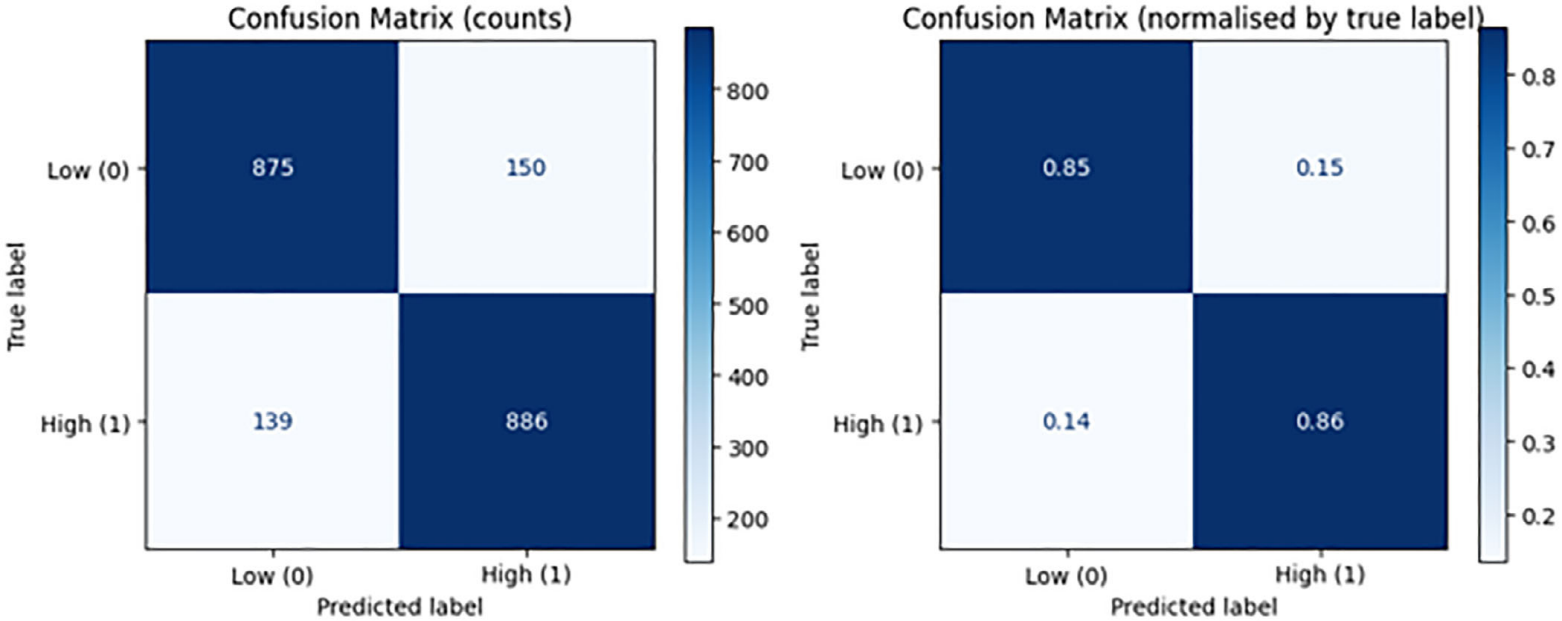
Patch‐level confusion matrices for the held‐out test set. Left: confusion matrix showing classification counts. Right: confusion matrix normalized by the true class. The test set included 1,025 high‐quality and 1,025 low‐quality CT/MR sub‐volume pairs.

For registration‐level evaluation, each of the 41 test patients contributed one high‐quality and one low‐quality registration condition, resulting in 82 registration‐level test cases. For the high‐quality cases, *μ* values ranged from 0.447 to 0.912, with a mean of 0.798. For the low‐quality cases, *μ* values ranged from 0.049 to 0.584, with a mean of 0.172. The individual registration‐level *μ* values for the held‐out test set are reported in Table 4, and their distribution relative to the fixed threshold is shown in Figure 7. Using the fixed validation‐derived *μ* threshold of 0.5871, the registration‐level test accuracy was 97.56%. The registration‐level ROC AUC was 0.999. The precision, sensitivity/recall, and F1‐score for the high‐quality class were 100.00%, 95.12%, and 97.50%, respectively, while the specificity for low‐quality registrations was 100.00%.

**TABLE 4:** Registration‐level mean probability (*μ*) values for the held‐out test set.

Case No.	High *μ*	Low *μ*
**1**	0.85	0.17
**2**	0.67	0.07
**3**	0.87	0.19
**4**	0.84	0.37
**5**	0.90	0.12
**6**	0.89	0.19
**7**	0.89	0.06
**8**	0.85	0.13
**9**	0.83	0.09
**10**	0.61	0.11
**11**	0.65	0.08
**12**	0.91	0.22
**13**	0.66	0.13
**14**	0.71	0.19
**15**	0.81	0.11
**16**	0.77	0.20
**17**	0.88	0.17
**18**	0.88	0.13
**19**	0.45	0.14
**20**	0.80	0.25
**21**	0.62	0.16
**22**	0.86	0.30
**23**	0.85	0.58
**24**	0.57	0.16
**25**	0.85	0.15
**26**	0.74	0.07
**27**	0.91	0.12
**28**	0.86	0.05
**29**	0.84	0.07
**30**	0.88	0.42
**31**	0.84	0.12
**32**	0.89	0.08
**33**	0.78	0.17
**34**	0.87	0.20
**35**	0.91	0.25
**36**	0.80	0.10
**37**	0.84	0.17
**38**	0.71	0.14
**39**	0.83	0.06
**40**	0.76	0.20
**41**	0.78	0.37

*Note*: Values are rounded to two decimal places for presentation. Classification decisions were performed using the exact *μ* values and the fixed validation‐derived threshold of 0.5871.

**FIGURE 7:**
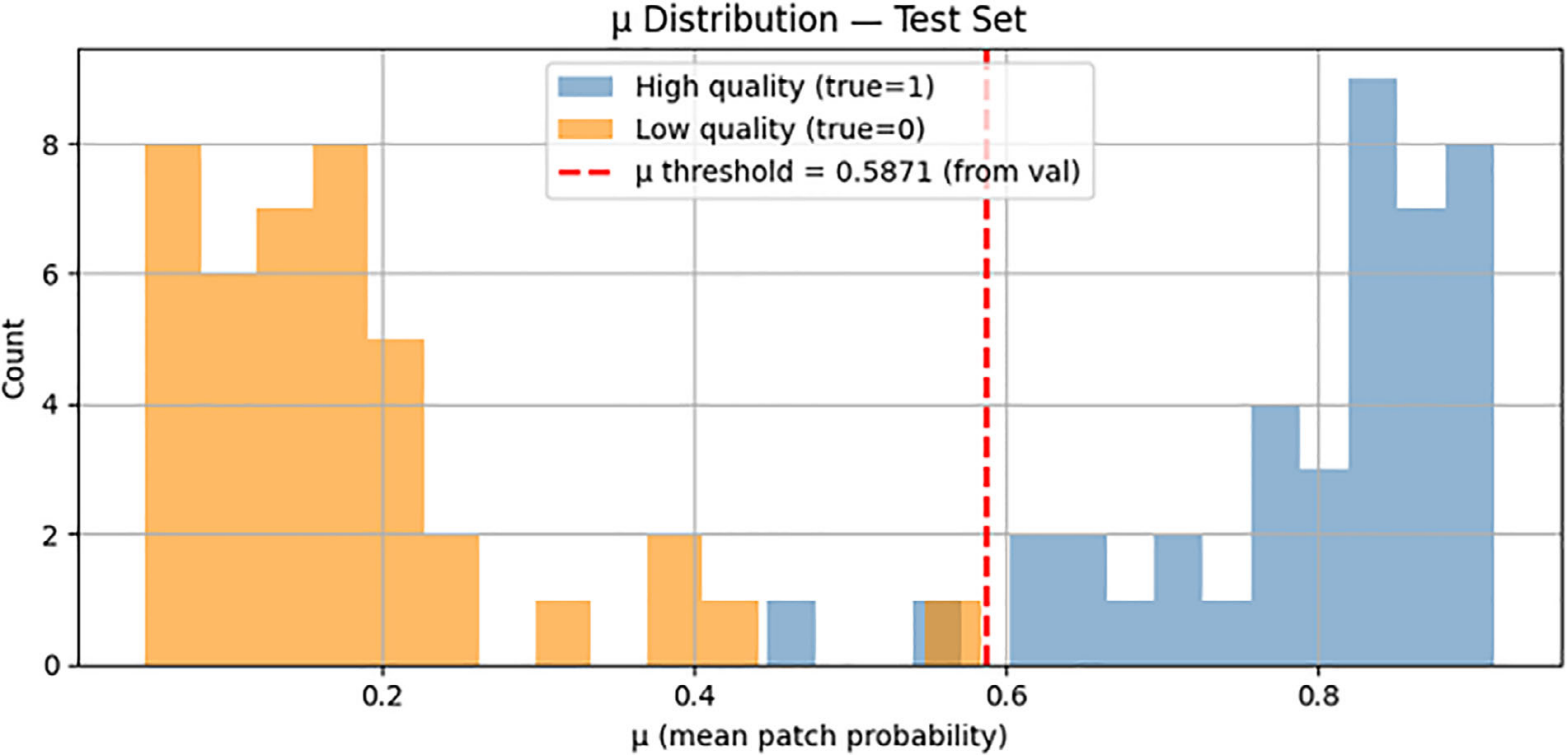
Held‐out test‐set distribution of the registration‐level mean probability (*μ*) for high‐quality and low‐quality cases. The dashed vertical line indicates the fixed validation‐derived *μ* threshold of 0.5871 applied without further adjustment.

Among the 41 high‐quality test cases, 39 were correctly accepted, and two were flagged for review. All 41 low‐quality cases were correctly flagged, with no low‐quality case incorrectly accepted. The overall registration‐level test performance using the fixed validation‐derived threshold is summarized in Table 5.

**TABLE 5:** Registration‐level performance on the held‐out test set using the fixed validation‐derived *μ* threshold of 0.5871.

Metric	Value
**Number of test patients**	41
**Registration‐level cases**	82
**Fixed *μ* threshold**	0.5871
**Registration‐level ROC AUC**	0.999
**Accuracy**	97.56%
**Precision for high‐quality class**	100%
**F1‐score for high‐quality class**	97.50%
**Sensitivity for high‐quality**	95.12%
**Specificity for low‐quality**	100%
**High‐quality correctly accepted**	39/41
**High‐quality flagged for review**	2/41
**Low‐quality correctly flagged**	41/41
**Low‐quality incorrectly accepted**	0/41

### 3.3 Clinical cases requiring correction

During routine clinical review, seven CT/MR registrations were not accepted at the initial physician review and required manual adjustment. The registrations before correction were retained as clinical low‐quality cases. After manual adjustment by the physician, the corrected and clinically approved registrations were retained as the corresponding high‐quality cases. The finalized model and the fixed validation‐derived *μ* threshold of 0.5871 were then applied to both registration conditions without further adjustment.

Across the seven patients, *μ* values for the corrected high‐quality registrations ranged from 0.569 to 0.772, with a mean of 0.690. For the initial registrations requiring correction, *μ* values ranged from 0.056 to 0.525, with a mean of 0.329. The patient‐specific *μ* values before and after physician correction are presented in Table 6 and illustrated in Figure 8.

**TABLE 6:** Registration‐level *μ* values for the seven independent clinical cases before and after physician correction.

Case No.	Initial registration *μ*	Initial classification	Corrected registration *μ*	Corrected classification
**1**	0.36	Low quality	0.57	Low quality
**2**	0.36	Low quality	0.73	High quality
**3**	0.06	Low quality	0.73	High quality
**4**	0.53	Low quality	0.77	High quality
**5**	0.39	Low quality	0.70	High quality
**6**	0.34	Low quality	0.69	High quality
**7**	0.27	Low quality	0.65	High quality

*Note*: Values are rounded to two decimal places for presentation. Classification decisions were performed using the exact *μ* values and the fixed validation‐derived threshold of 0.5871. Initial registrations were those requiring physician correction, whereas corrected registrations were subsequently clinically approved.

**FIGURE 8:**
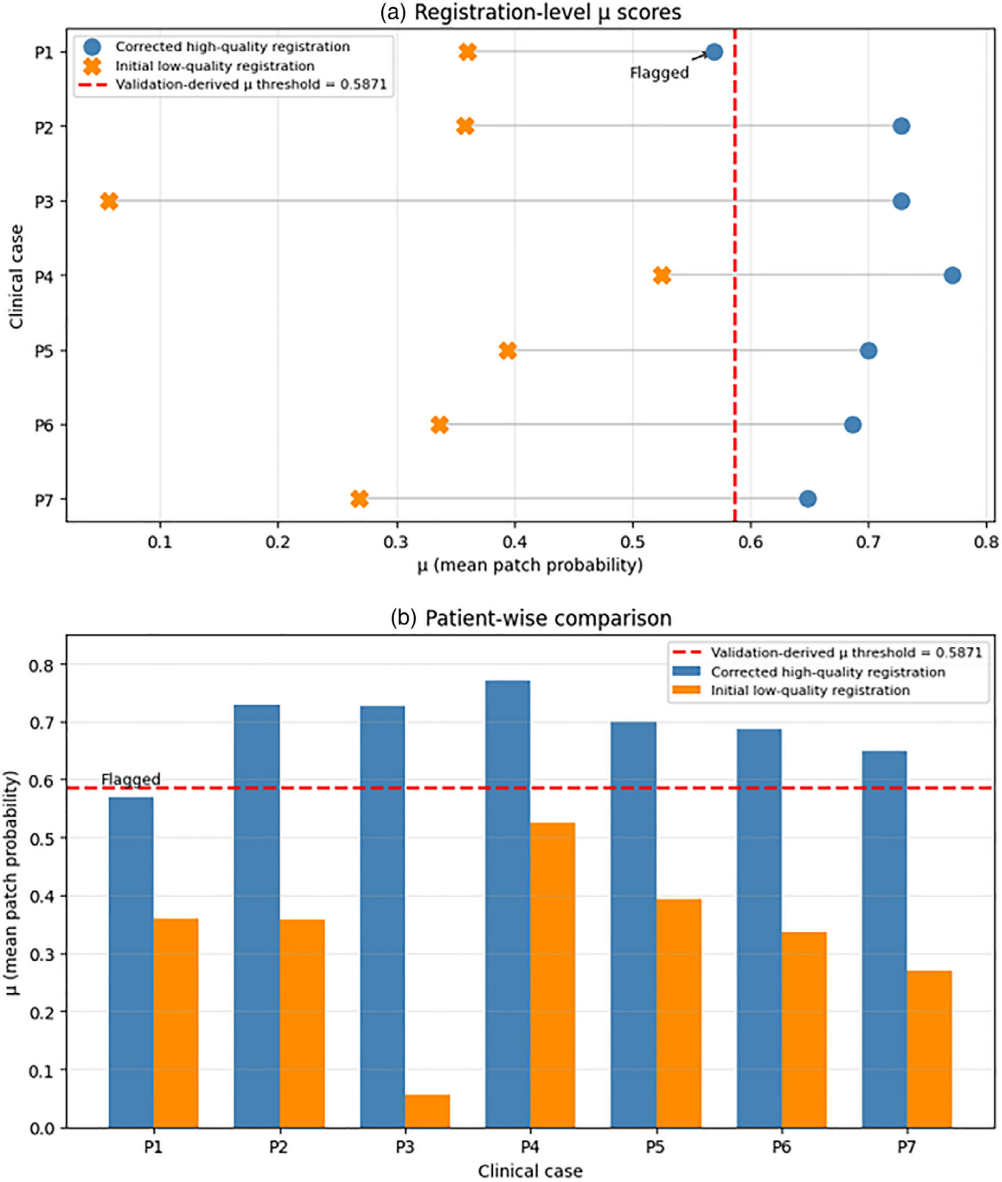
Registration‐level *μ* scores for the seven independent clinical cases evaluated before and after physician correction. (A) *μ* values for the initial registrations requiring correction and the corresponding corrected and clinically approved registrations. (B) Patient‐wise comparison of *μ* values for the two registration conditions. The dashed line indicates the fixed validation‐derived *μ* threshold of 0.5871.

Using the fixed *μ* threshold, all seven initial registrations requiring correction were correctly classified as low quality. Six of the seven corrected and clinically approved registrations were correctly classified as high quality, while one was flagged for review with *μ* = 0.570. Overall, 13 of the 14 registration conditions were correctly classified, corresponding to an accuracy of 92.86%. For the high‐quality class, sensitivity was 85.71%, precision was 100%, and the F1‐score was 92.31%, while specificity for the low‐quality registrations was 100%.

## 4 DISCUSSION

In this study, a 3D CNN‐based QA method was developed to assess rigid brain CT/MR registration quality using paired sub‐volumes. The model was trained to classify each CT/MR sub‐volume pair as high quality or low quality, and the sub‐volume predictions were then combined into a registration‐level mean probability score (*μ*). At the patch level, the model achieved a validation accuracy of 0.858 and a validation F1‐score of 0.861. On the held‐out test set, the patch‐level accuracy was 85.90%, with an ROC AUC of 0.929 and an average precision of 0.920. When the validation‐derived *μ* threshold was applied to the held‐out test set, the registration‐level accuracy was 97.56%, with a registration‐level AUC of 0.999.

The use of sub‐volumes was important in this work because full‐volume 3D CT/MR input can be limited by GPU memory and may also include many regions that are less useful for rigid registration QA. By using paired CT/MR sub‐volumes, the model was able to focus on local anatomical regions while still allowing registration quality to be assessed from multiple sampled locations. This approach also increased the number of training samples while keeping the split patient‐wise to avoid data leakage between training, validation, and testing.

The registration‐level mean probability *μ* was used to summarize the predictions obtained from the 25 sampled sub‐volumes. This was needed because the CNN provides one probability value for each extracted sub‐volume, whereas the clinical assessment concerns the complete CT/MR registration. Averaging the local predictions provided a single registration‐level score representing the overall model confidence across the sampled regions. The difference between the patch‐level and registration‐level results suggests that combining predictions across multiple anatomical locations can provide a more stable assessment of the overall registration than relying on individual sub‐volumes alone.

The validation set was used to define the *μ* threshold before final testing, rather than to optimize the threshold using the test data. ROC analysis of the validation registration‐level scores resulted in a threshold of 0.5871 using the Youden index. This threshold was then fixed and applied to the held‐out test set without further adjustment. Therefore, the reported test performance reflects the application of a preselected decision threshold rather than a threshold optimized after observing the test results.

Low‐quality examples were generated by applying one six‐degree‐of‐freedom rigid perturbation to the complete MR volume for each patient before sub‐volume extraction. Rotations and translations were randomly sampled within ± 5° and ± 5 mm, respectively. An important aspect of the preprocessing framework was the use of a common CT‐derived physical grid and physical‐coordinate mapping between CT and MR images. This avoided assuming that identical voxel indices represented the same physical anatomical locations across modalities. In addition, the synthetic rigid perturbation was incorporated directly into the resampling of the original MR image onto the common grid, so that both high‐ and low‐quality MR images underwent a single linear resampling operation before preprocessing and patch extraction. This reduced differences in interpolation history between the two classes and therefore reduced the possibility that classification was driven by class‐specific resampling artifacts rather than registration misalignment itself. Although this approach does not eliminate all potential effects associated with synthetic perturbations, it provides a more balanced framework for generating low‐quality registration examples.

The independent test results showed strong separation between the high‐ and low‐quality registration‐level *μ* values. Among the 41 high‐quality registrations, 39 were correctly accepted and two were flagged for review. All 41 synthetically perturbed low‐quality registrations were correctly flagged, with no low‐quality case incorrectly accepted using the fixed threshold. The two misclassified registrations were therefore clinically approved high‐quality cases that received a conservative low‐quality classification. In a QA screening setting, such errors would lead to additional manual inspection rather than automatic acceptance of a potentially inaccurate registration. Nevertheless, the proposed score should be considered a screening aid rather than a replacement for clinical review.

The additional independent clinical evaluation provided a complementary assessment using seven patients who were separate from the 202‐patient primary cohort and were evaluated after model development and threshold selection had been completed. All seven initial registrations that were not accepted by the reviewing physician and required manual adjustment were correctly classified as low‐quality using the fixed validation‐derived *μ* threshold. After physician correction, six of the seven clinically approved registrations were classified as high quality, resulting in an overall accuracy of 92.86% across the 14 registration conditions. Although the number of clinical cases was limited, these findings provide preliminary evidence that the model can identify registration problems encountered during routine clinical review, supporting evaluation beyond synthetically generated perturbations.

The single misclassified clinical case was a corrected and clinically approved registration with *μ* = 0.570, which was close to the predefined threshold of 0.5871. This error pattern was consistent with the validation and held‐out test results, in which the few registration‐level misclassifications also involved high‐quality registrations being flagged for review, while all low‐quality registrations were correctly identified. Specifically, one of 33 high‐quality validation registrations and two of 41 high‐quality test registrations were flagged, whereas all low‐quality registrations in both sets were correctly classified. This suggests that, at the selected threshold, the model tended toward conservative flagging rather than incorrectly accepting low‐quality registrations. Such behavior may be preferable for a QA screening tool, although the limited number of clinical cases requires cautious interpretation.

The training and validation curves showed that training performance continued to improve while validation performance reached its best level earlier in training and fluctuated. Early stopping and restoration of the best‐performing validation weights were therefore used to limit the effect of further overfitting. The relatively similar final patch‐level performance on the validation and held‐out test sets also suggests that the selected model retained comparable classification performance on unseen patients.

The skull‐based sampling strategy was selected because the study focused on rigid brain CT/MR registration. The CT bone mask was used to identify stable landmark locations for sub‐volume extraction, since the skull is a rigid structure and provides reliable anatomical reference points for brain registration. The additional exclusion of the inferior portion of the image reduced sampling from the mandible and cervical vertebrae. However, because the present work focused mainly on skull‐based sampling, the model may be less sensitive to localized soft‐tissue mismatches away from the sampled cranial structures. Future work could investigate combined skull and soft‐tissue sampling, particularly for applications involving deformable registration.

Compared with segmentation‐based registration QA methods, the proposed approach does not require manual contours or predefined segmentation masks. Instead, it evaluates an existing rigid CT/MR registration directly from paired image sub‐volumes. This may be useful in radiotherapy planning workflows in which the registration has already been performed and the practical objective is to identify registrations that may require additional inspection. The registration‐level *μ* score is therefore intended to provide an additional quantitative screening measure while keeping the final decision within the clinical review process.

The findings of this study should be interpreted in the context of the controlled study design. The primary model development and evaluation relied on synthetically generated rigid perturbations. Although an additional independent set of seven clinical cases requiring physician correction was evaluated, this cohort was limited in size and may not represent the full range of registration problems encountered in clinical practice. The synthetic perturbations were applied to the complete MR volume and incorporated directly into the common‐grid resampling procedure; however, synthetic errors may not fully reproduce all clinically encountered registration failures. In addition, this study focused on rigid brain CT/MR registration in SRS and SRT cases and did not specifically evaluate major anatomical changes, severe deformation, or other anatomical sites. The derived *μ* threshold should therefore not be assumed to apply directly to other registration tasks without further evaluation. The study was also not designed to estimate the prevalence of clinically unacceptable registrations in routine practice, and prevalence‐dependent measures such as positive predictive value and negative predictive value were therefore not calculated. Further work should include a larger independent clinical cohort and investigate the effect of the number and spatial distribution of sampled sub‐volumes on registration‐level performance.

Despite these considerations, the results demonstrate that a 3D CNN using paired CT/MR sub‐volumes can distinguish high‐ and low‐quality rigid registrations and that averaging the local predictions into a registration‐level *μ* score provides strong separation in the held‐out test set. The additional independent clinical cases further support the potential of the method to identify registrations requiring correction during routine clinical review. The method may therefore serve as a quantitative screening tool to support clinical review of rigid brain CT/MR registrations.

## 5 CONCLUSIONS

In this study, a 3D CNN‐based method was developed for QA of rigid brain CT/MR registration using paired CT/MR sub‐volumes as a two‐channel input. The model classified local registration quality, and the resulting sub‐volume probabilities were summarized using the registration‐level mean probability (*μ*).

The *μ* threshold was selected using the validation set and applied to the held‐out test set without further adjustment. The model achieved a patch‐level test accuracy of 85.90% and an ROC AUC of 0.929, while registration‐level evaluation using *μ* achieved an accuracy of 97.56% and an AUC of 0.999. All synthetically perturbed low‐quality registrations in the held‐out test set were correctly flagged.

In the additional clinical evaluation, all seven initial registrations that required physician correction were correctly flagged as low quality, while six of the seven corrected and clinically approved registrations were classified as high quality.

These results indicate that the proposed approach can provide a useful quantitative screening measure for rigid brain CT/MR registration QA while maintaining clinical review as the final decision step.

## CONFLICT OF INTEREST STATEMENT

The authors declare no conflicts of interest.

## ETHICAL APPROVAL

This retrospective study used fully anonymized imaging data collected as part of a routine clinical radiotherapy workflow, including institutional CT/MR scans and externally acquired MR scans that were clinically accepted and registered to institutional CT scans for treatment planning. According to institutional guidance, formal ethics approval was not required because no identifiable patient information was used and no intervention was performed.

## CONSENT TO PARTICIPATE

Informed consent was not required due to the retrospective nature of the study and the use of fully anonymized imaging data.

## CONSENT FOR PUBLICATION

Not applicable. The manuscript does not include identifiable individual patient data.

## Data Availability

The imaging datasets used in this study contain de‐identified and preprocessed data may be made available from the corresponding author upon reasonable request. The custom code developed for data preprocessing, model training, and evaluation is available from the corresponding author upon reasonable request.
